# Drug response profiles in patient-derived cancer cells across histological subtypes of ovarian cancer: real-time therapy tailoring for a patient with low-grade serous carcinoma

**DOI:** 10.1038/s41416-022-02067-z

**Published:** 2022-12-07

**Authors:** Astrid Murumägi, Daniela Ungureanu, Suleiman Khan, Mariliina Arjama, Katja Välimäki, Aleksandr Ianevski, Philipp Ianevski, Rebecka Bergström, Alice Dini, Anna Kanerva, Riitta Koivisto-Korander, Johanna Tapper, Heini Lassus, Mikko Loukovaara, Andrus Mägi, Akira Hirasawa, Daisuke Aoki, Vilja Pietiäinen, Teijo Pellinen, Ralf Bützow, Tero Aittokallio, Olli Kallioniemi

**Affiliations:** 1 Institute for Molecular Medicine Finland (FIMM), Helsinki Institute of Life Science (HiLIFE), University of Helsinki, Helsinki, Finland; 2 Applied Tumor Genomics Research Program, Faculty of Medicine, University of Helsinki, Helsinki, Finland; 3 Faculty of Biochemistry and Molecular Medicine, University of Oulu, Oulu, Finland; 4 Helsinki Institute for Information Technology (HIIT), Department of Computer Science, Aalto University, Espoo, Finland; 5 Department of Medical Epidemiology and Biostatistics, Karolinska Institutet, Solna, Sweden; 6 Department of Obstetrics and Gynecology, University of Helsinki and Helsinki University Hospital, Helsinki, Finland; 7 Tartu University Hospital, Tartu, Estonia; 8 Department of Clinical Genomic Medicine, Graduate School of Medicine, Dentistry and Pharmaceutical Sciences, Okayama University, Okayama, Japan; 9 Department of Obstetrics and Gynecology, Keio University School of Medicine, Tokyo, Japan; 10 iCAN Digital Precision Cancer Medicine Flagship, University of Helsinki, Helsinki, Finland; 11 Department of Pathology, University of Helsinki and Helsinki University Hospital, Helsinki, Finland; 12 Institute for Cancer Research, Department of Cancer Genetics, Oslo University Hospital, Oslo, Norway; 13 Centre for Biostatistics and Epidemiology (OCBE), Faculty of Medicine, University of Oslo, Oslo, Norway; 14 Science for Life Laboratory (SciLifeLab), Department of Oncology and Pathology, Karolinska Institutet, Solna, Sweden

**Keywords:** Ovarian cancer, Systems biology

## Abstract

Many efforts are underway to develop novel therapies against the aggressive high-grade serous ovarian cancers (HGSOCs), while our understanding of treatment options for low-grade (LGSOC) or mucinous (MUCOC) of ovarian malignancies is not developing as well. We describe here a functional precision oncology (fPO) strategy in epithelial ovarian cancers (EOC), which involves high-throughput drug testing of patient-derived ovarian cancer cells (PDCs) with a library of 526 oncology drugs, combined with genomic and transcriptomic profiling. HGSOC, LGSOC and MUCOC PDCs had statistically different overall drug response profiles, with LGSOCs responding better to targeted inhibitors than HGSOCs. We identified several subtype-specific drug responses, such as LGSOC PDCs showing high sensitivity to MDM2, ERBB2/EGFR inhibitors, MUCOC PDCs to MEK inhibitors, whereas HGSOCs showed strongest effects with CHK1 inhibitors and SMAC mimetics. We also explored several drug combinations and found that the dual inhibition of MEK and SHP2 was synergistic in MAPK-driven EOCs. We describe a clinical case study, where real-time fPO analysis of samples from a patient with metastatic, chemorefractory LGSOC with a *CLU-NRG1* fusion guided clinical therapy selection. fPO-tailored therapy with afatinib, followed by trastuzumab and pertuzumab, successfully reduced tumour burden and blocked disease progression over a five-year period. In summary, fPO is a powerful approach for the identification of systematic drug response differences across EOC subtypes, as well as to highlight patient-specific drug regimens that could help to optimise therapies to individual patients in the future.

## Introduction

Epithelial ovarian cancer (EOC) is the most lethal gynaecological cancer, with a 5-year overall survival rate of about 30–40%, which have not improved much over the last 30 years, despite advances in surgery, imaging technologies, and introduction of new targeted therapies, such as PARP inhibitors and anti-angiogenic drugs such as bevacizumab [1–4]. Current standard-of-care for patients with EOC involves debulking and platinum-based chemotherapy, usually in combination with a taxane, with some patients receiving PARP inhibitors. However, many patients will ultimately relapse and develop chemotherapy-resistant advanced disease, at which point treatment options are mostly palliative.

EOC is classified into several histological subtypes. For instance, low-grade serous ovarian cancer (LGSOC), endometrioid, mucinous (MUCOC) and clear cell, are often detected at an early stage (FIGO I–II) and have more stable genomes, and are characterised by specific somatic mutations: LGSOC with *KRAS and BRAF*, endometrioid with *PTEN and PI3KCA*, MUCOC with *TP53* and *KRAS*, and clear cell with *PI3KCA and ARID1A*. LGSOCs are often resistant to platinum-based chemotherapy [5]. On the other hand, high-grade serous ovarian cancer (HGSOC), high-grade endometrioid, undifferentiated cancers and carcinosarcomas often have high genomic instability, mutant *TP53*, defects in homologous recombination (HR) repair, mutations or impairment in *BRCA1/2*, and extensive copy number aberrations [6]. Although these EOC subtypes are initially more responsive to platinum-based chemotherapy, they tend to progress to therapy resistance. The majority of deaths from EOC are caused by the HGSOCs. HR deficiency is a key determinant of sensitivity to platinum as well as to poly(adenosine diphosphate–ribose) polymerase (PARP) inhibitors such as olaparib [7, 8].

Most precision oncology programmes to date are based on tailoring treatments based on genome sequencing data, and hence they often focus on blocking activated driver oncogenes and their signalling [9, 10]. While this has been successful in some cases, there are many cancers where druggable oncogenic drivers are seldom found. Furthermore, not all tumours that are genetically predicted to respond to targeted treatments actually show responses, as illustrated by the results from the National Cancer Institute Molecular Analysis for Therapy Choice (NCI-MATCH) trial [11]. Functional drug testing of patient-derived cancer cells (PDCs), or patient-derived organoids, provides a unique opportunity to directly identify effective drugs and drug combinations, even for tumours that lack actionable driver mutations [12, 13]. Personalising cancer therapy based on functional testing of PDCs has shown great promise in identifying clinically translatable treatments in haematological malignancies [14–17] as well as in some solid tumours [13, 18, 19]. This is particularly exciting for solid tumours, where it is more challenging to establish representative ex vivo models for functional precision oncology purposes.

Here, we introduce a functional precision oncology approach for the real-time identification of potential therapeutics for EOC patients in a clinically actionable time frame, based on the establishment and functional drug sensitivity testing of 16 representative PDCs derived from 13 HGSOC, LGSOC and MUCOC patients. We first studied to what extent do these three tumour types differ from each other, not only in terms of genomic and transcriptomic profiles, but also based on their global drug response profile. The second aim was to functionally identify novel therapeutic opportunities for individual EOC patients. The 16 PDCs were tested with a library of up to 526 approved drugs and emerging oncology compounds. Based on integrated data from molecular profiling and drug sensitivity testing, we identified (i) distinct subtype-specific drug response differences, (ii) functional evidence of dependency of EOC on specific pathways, (iii) previously undescribed drug repositioning opportunities, and (iv) EOC cases where specific drug combinations were effective. A patient with a metastatic LGSOC was treated according to predictions from the integrated analyses, resulting in tumour reduction and disease control over a 5-year period. Overall, this study demonstrates the potential of functional precision oncology to tailor therapeutic opportunities for EOC.

## Materials and methods

### Patient samples and establishment of patient-derived cancer cell cultures

All patient material and clinical data were obtained upon informed consent, under Institutional Ethical Review Board-approved protocol and in accordance with the Declaration of Helsinki. Patients’ clinical characteristics are summarised in Table 1 and Supplementary Table S1A. Patients received treatment either at the Helsinki University Central Hospital, Finland or at the Tartu University Hospital, Estonia. All except one patient (FMOC25) had received either neoadjuvant treatment or standard chemotherapy before the sample collection. From two LGSOC cases, multiple samples were obtained to establish PDCs at different stages of tumour progression. Biopsies were minced into smaller pieces using a sterile scalpel and underwent enzymatic digestion using a Tumour Dissociation kit (Miltenyi Biotec) and gentle MACS Dissociator (Miltenyi Biotec) to obtain single-cell suspension. After centrifugation at 300 × *g*, both tumour-derived cells and ascites underwent red blood cell removal using Red Blood Cell Lysis Solution according to the manufacturer’s protocol (Miltenyi Biotec). All HGSOC and MUCOC PDCs, except FMOC04, were established using the protocol described by Liu et al. [20, 21]. Briefly, patients’ cancer cells were co-cultured with irradiated mouse 3T3 fibroblast feeder cells in a culture medium supplemented with Rho-kinase (ROCK) inhibitor Y-27632 (Enzo Life Science). FMOC04 and LGSOC PDCs were established in a serum-free stem cell media DMEM-F12 supplemented with 20 ng/ml EGF (Corning), 10 ng/ml FGF (Invitrogen), B27 (Thermo Fisher Scientific) and primocin (Invivogen). One of the biggest challenges we faced was the presence of fast dividing stromal cells during the development of the models. Cancer-associated fibroblasts that would have otherwise overgrown the epithelial cancer cells were removed by double trypsination or by selection with anti-fibroblast microbeads and magnetic MACS Separator (Miltenyi Biotec). Our success rate was 53% with both culturing protocols and across all three OC subtypes (50% for HGSOC, 60% for LGSOC and 50% for MUCOC, respectively). The proliferation rate of individual PDCs and time between the surgery and drug testing experiment are presented in the Supplementary Table S1B. The OC cell line Kuramochi was obtained from JCRB Cell Bank and cultured in the recommended media. Primary PDCs and OC cell line were maintained at 37 °C with 5% CO_2_ and passaged weekly. All experiments were performed with early passage cells (under passage 10).

**Table 1:** EOC patient cases and respective PDCs included in the study.

Patient ID	Histological OC subtype	Disease stage	Sample type	Key oncogenic aberrations	Selected drug efficacies seen in PDCs
FMOC04	HGSOC	Recurrent (peritoneal metastases)	Ascites	TP53 p.R175H, CCNE1 Amp, MECOM Amp, PIK3CA gain	AZD1775 (Wee1i), prexasertib (Chki)
FMOC09	HGSOC	Primary	Tissue	TP53 p.R283P, CCNE1 Amp, MECOM Amp, PIK3CA Amp, FNBP4-PTPMT1 fusion	Anagrelide (PDE-3i), dasatinib (multikinasei), afatinib (ERBB2/EGFRi), prexasertib (Chki)
FMOC11	HGSOC	Primary	Tissue	TP53 frameshift, MYC Amp, KRAS Amp, PIK3CA gain, ING5-THAP4 fusion, ROCK1-SS18 fusion	AZD1775 (Wee1i), prexasertib (Chki), poziotinib (pan-ERBBi), dasatinib (multikinasei)
FMOC14	HGSOC	Peritoneal metastases	Ascites	TP53 p.A161T, broad CNV changes incl. PIK3CA gain	AZD1775 (Wee1i), prexasertib (Chki), omipalisib (PI3K/mTORi), AZD8055 (mTORi)
FMOC24	HGSOC	Primary, progressive	Ascites	TP53 p.R273C, CCNE1 and KRAS Amp, PIK3CA gain, RCC1-UBE2D2 fusion	NVP-LCL161 & birinapant (SMAC mimetic), prexaserib (Chki), navitoclax (BCL-2i)
FMOC02 (FMOC02_1 FMOC02_2 FMOC02_3)	LGSOC	Recurrent (peritoneal metastases)	(1) Ascites (FMOC02_1) (2) Needle biopsy (FMOC02_2) (3) Ascites (FMOC02_3)	TP53 wt, CLU-NRG1 fusion, CDKN2A homozygous loss	Poziotinib & dacomitinib (pan-ERBBi), afatinib (ERBB2/EGFRi), AZD8055 (mTORi), SCH772984 (ERK1/2i), AMG-232 (MDM2i)
FMOC17	LGSOC	Primary	Tissue	TP53 wt, CDKN2A homozygous loss	Poziotinib (pan-ERBBi), dasatinib (multikinasei), SCH772984 (ERK1/2i), AMG-232 (MDM2i)
FMOC25 (FMOC25_1 FMOC25_2)	LGSOC	Primary, progressive	(1) Tissue (FMOC25_1) (2) Tissue (FMOC25_2)	TP53 wt, TACSTD2-OMA1 fusion, CDKN2A homozygous loss (FMOC25_2)	AZD8055 (mTORi), NVP-BGT226 & omipalisib (PI3K/mTORi), SCH772984 (ERK1/2i), AMG-232 (MDM2i)
FMOC27	LGSOC	Primary	Tissue	TP53 wt, CDKN2A homozygous loss	Poziotinib & dacomitinib (pan-ERBBi), afatinib (ERBB2/EGFRi), SCH772984 (ERK1/2i), AMG-232 (MDM2i)
FMOC28	LGSOC	Primary	Tissue	TP53 wt, CDKN2A homozygous loss	AZD8055 (mTORi), poziotinib (pan-ERBBi), NVP-BGT226 (PI3K/mTORi), AMG-232 (MDM2i)
FMOC03	MUCOC	Primary	Tissue	TP53 p.G244V, WT1 p.H469Y, ERBB2 Amp.	Poziotinib (pan-ERBBi), SCH772984 (ERK1/2i), afatinib (ERBB2/EGFRi)
FMOC06	MUCOC	Recurrent	Ascites	TP53 p.S215N, KRAS p.G12D, CDKN2A & MTAP homozygous loss	Omipalisib (PI3K/mTORi), AZD8055 (mTORi), SCH772984 (ERK1/2i), selumetinib & trametinib (MEKi)
FMOC22	MUCOC	Primary	Ascites	TP53 p.D281H, KRAS p.G12V, CDKN2A & MTAP homozygous loss	Prexasertib (Chki), AZD8055 (mTORi), SCH772984 (ERK1/2i)

In addition to histological EOC subtype and disease stage, the key oncogenic aberrations are presented for each patient case along with the selective effective targeted drugs based on the DSRT platform. More detailed clinical characteristics for patient cases are presented in the Supplementary Table S1A. *HGSOC* high-grade serous ovarian carcinoma, *LGSOC* low-grade serous ovarian carcinoma, *MUCOC* mucinous ovarian cancer.

### Antibodies and reagents

The following antibodies EGFR (#4267), p-EGFR (#3777), ERBB2 (#2242), p-ERBB2 (#2243), ERBB3 (#4754), p-ERBB3 (#4791), ERK1/2 (#4696), p-ERK1/2 (T202/Y204, #9101), AKT (#9272) and p-AKT (#6942) were purchased from Cell Signaling Technology. β-tubulin antibody (#sc-166729) was purchased from Santa Cruz Biotech and actin antibody (#A2066) from Sigma-Aldrich. Afatinib was purchased from Selleck Chemicals LLC; erlotinib, selumetinib and SHP099 from MedChem Express; and trametinib from ChemieTek. Therapeutic antibodies trastuzumab and pertuzumab were obtained from Roche, Finland.

### Drug sensitivity and resistance testing (DSRT)

DSRT was performed with PDCs with drug libraries containing up to 526 approved drugs and investigational compounds as described previously [22]. In addition, available drug testing data obtained with the healthy bone marrow derived mononuclear cells were used as controls [16]. Bone marrow aspirates from healthy donors (*n* = 2) were obtained after an informed consent and were collected at the Helsinki University Hospital following protocols approved by a local ethics committee and in accordance with the Declaration of Helsinki. The drug library was updated one time during the study and the list of compounds in both libraries and list of samples screened with individual libraries is presented in Supplementary Tables S1C–S1E. Briefly, the drugs were dissolved in 100% dimethyl sulfoxide (DMSO) or water and plated in five concentrations covering a 10,000-fold range on 384-well flat clear bottom tissue culture treated microplates (Corning) using an Echo 550 acoustic dispenser (Labcyte). Cells were dispensed on pre-drugged plates with the Multidrop dispenser (Thermo Fisher Scientific) and incubated for 72 h at 37 °C and 5% CO_2_. Cell viability was measured with CellTiter-Glo Cell Viability Assay (Promega). The assay was carried out similarly for measuring the drug responses in 3D culture conditions, except PDCs were seeded on ultra-low attachment 384-well round bottom cell culture plates (Corning) pre-plated with drugs in nine increasing concentrations. Drug synergy testing was performed using 7 × 7 drug concentration matrix (including negative (DMSO) and positive (benzethonium chloride) controls and 7 concentrations per drug and combination), where cell viability was assessed with CellTiter-Glo Cell Viability Assay (Promega), similarly as before [23].

### Drug sensitivity scoring (DSS)

To quantify the compound responses, drug sensitivity score (DSS) was calculated for each PDC and compound separately [24]. DSS calculates a partial area under the dose-response curve (AUC), using an activity window from 10 to 100% and a dose-window either from the minimum concentration tested or from the concentration where the %inhibition reaches 10%. DSS3 metric was used, which divides the partial AUC by the logarithm of the upper asymptote of the logistic curve. Higher levels of DSS indicate higher sensitivity to the compound. DSS has been shown to provide a reproducible drug response metric across multiple screening sites in pan-cancer cell line analyses, when compared to other drug response metrics, such as activity area or IC_50_ [25].

### Colony forming assay (CFA)

Cells (5 × 10^3^) were seeded in triplicates in 12-well plate. After 24 h, the cells were treated with vehicle (DMSO) or the indicated single drugs or drug combinations. Medium and drugs were refreshed every 3 days for 14 days. Colonies were fixed with methanol/glacial acetic acid (7:1) and stained with 0.5% of crystal violet. After washing with PBS, plates were air dried overnight and scanned. Representative images of confluency where taken with the IncuCyte HD (Essen Bioscience) and colonies were quantified with ImageJ using the ColonyArea plugin [26].

### Immunohistochemistry (IHC)

For IHC analysis, formalin-fixed paraffin-embedded (FFPE) blocks were cut as 3.5 μm sections and stained for antibodies against pan-cytokeratin (Sigma-Aldrich, C-11, #C2931), PAX8 (ProteinTech, #10336-1-AP), TP53 (Cell Signaling Technology, #48818), WT1 (Cell Signaling Technology, #83535), p-ERBB2 (Cell Signaling Technology, #2243), p-EGFR (Cell Signaling Technology, #3777), and p-ERBB3 (Cell Signaling Technology, #4791) according to standard procedures. The stained sections were scanned with a high-resolution whole-slide scanner (Pannoramic 250 Flash III, 3DHISTECH) with a ×20 objective.

### Next-generation DNA sequencing

Genomic DNA was isolated from EOC tissue samples, PDC and germline control blood cells using the DNeasy Blood and Tissue kit (Qiagen) according to the manufacturer’s protocol. Genomic DNA concentration and purity was measured with Qubit 2.0 Fluorometer (Thermo Fisher Scientific). Exome sequencing was performed for samples FMOC03 (both original tumour tissue and PDC), FMOC04 (PDC), FMOC06_1 (both original tumour tissue and PDC), FMOC11 (both original tumour tissue and PDC), FMOC14 (both original tumour tissue and PDC) with 3 μg of genomic DNA using NimbleGen SeqCap EZ Human Exome v2.0 kit (Roche NimbleGen). The mutation and copy number analysis were carried out as described previously [27]. Sequencing reads were aligned and processed through variant calling pipeline [27]. Whole-genome sequencing was performed for FMOC09 (both original tumour tissue and PDC) as described earlier [27]. The cancer panel sequencing was performed for FMOC02_1 (PDC), FMOC02_2 (PDC), FMOC02_3 (PDC), FMOC17 (PDC), FMOC24 (original ascites and PDC), FMOC25_1 (both original tumour tissue and PDC), FMOC25_2 (both original tumour tissue and PDC) and FMOC27 (both original tumour tissue and PDC). For the library preparation, in‐solution hybridisation–based capture, and sequencing were performed as previously described [28]. Briefly, an average of 50 ng of DNA was used for library preparation (ThruPLEX Plasma Seq; Rubicon Genomics). Capture was performed with a custom pan-cancer panel (Roche NimbleGen) which enables detection of somatic alterations in coding sequence (289 genes) and genome‐wide copy number variants (CNVs). The captured libraries were sequenced in rapid mode on the HiSeq 2500 instrument (Illumina). Downstream bioinformatics, including basic quality control and identification of mutations and CNVs was performed as previously described [28]. Copy number values and segmentation were visualised in the Integrative Genomics Viewer (Broad Institute, USA).

### RNA-sequencing

Total RNA was isolated from PDCs using RNeasy kit (Qiagen). Quantity and quality of the RNA samples were assessed by Qubit (Thermo Fisher Scientific) and Bioanalyzer (Agilent Technologies). RNA with an RNA integrity number (RIN) > 8 was used for subsequent analysis. Libraries were multiplexed and paired-end sequencing was performed with Illumina HiSeq system (Illumina). RNA-sequencing data were analysed as previously described [29]. Using the normalised log-transformed CPM (count per million) expression values for 19,686 genes, hierarchical clustering was performed to generate a sample-to-sample cluster map of total gene expression correlations with the SciPy Python library [30] which was then visualised using the Seaborn Python library (10.5281/zenodo.1313201). Hierarchical clustering was performed for unique sets of 39 genes linked to the RAS pathway. Distances were calculated with the Euclidean distance metric and the Ward variance minimisation algorithm was used to cluster genes and samples, based on log-transformed CPM expression values in 11 PDCs, with the SciPy Python library [30]. Expression values were normalised on a zero-to-one scale, and the derived clusters were then visualised using the Seaborn Python library as described above. Fusion genes were predicted using the Fusion Catcher tool [31].

### RT-PCR and Sanger sequencing

Three micrograms of total RNA was used for the first-strand cDNA synthesis using the High Capacity cDNA Reverse Transcription Kit (Applied Biosystems). RT-PCR was performed with Phusion Flash High-Fidelity PCR master mix (Thermo Fisher Scientific). Verification of fusion genes was carried out at the FIMM Technology Centre Sequencing Unit by capillary sequencing. Primer sequences are listed in Supplementary Table S1F.

### Western blotting

Cells were washed twice in cold PBS and lysed with lysis buffer (50 mM Tris-HCl pH 7.5, 10% glycerol, 150 mM NaCl, 1 mM EDTA, 1% Triton-x-100, 50 mM NaF) supplemented with protease and phosphatase inhibitor cocktails (Biotool). Lysates were resolved by SDS–PAGE and transferred to nitrocellulose membranes. After blocking in 5% BSA, blots were incubated with the indicated primary antibodies overnight at 4 °C. After primary antibodies IRDye secondary antibodies IRDye 680RD Donkey anti-Goat IgG, IRDye 800CW Donkey anti-Mouse IgG or IRDye 680RD Donkey anti-Rabbit IgG (LI-COR) were used at 1:10,000 dilution and blots were scanned with Odyssey CLx Imaging System (LI-COR) and images were processed with Image Studio Lite (LI-COR).

### Target addiction scoring (TAS)

Target addiction scoring (TAS) is an experimental-computational target deconvolution method, which makes use of polypharmacological effects of compounds to integrate the drug sensitivity and selectivity profiles through systems-wide interconnection networks between drugs and their targets, including both primary protein targets as well as potent off-targets [32, 33]. More specifically, for a given kinase target *t*, *TAS*_*t*_ was calculated by averaging the observed drug response (DSS_*i*_) over those *n*_*t*_ inhibitors that target the protein *t*. Mathematically, TAS defines a transformation between the spaces spanned by the compounds and their targets, which maps observed drug responses to the underlying target addictions as

$${{{{{\rm{TAS}}}}}}_t = \mathop {\sum }\limits_{i = 1}^{n_t} \frac{{{{{{{\rm{DSS}}}}}}_i}}{{n_t}}.$$

TAS is an individualised approach in the sense that it uses the drug sensitivity profile from a given PDC cells, screened against a library of drugs using DSRT, and then transforms the observed functional response profile into a sample-specific target addiction profile, through the information encoded in the spectrum of protein targets of the compounds. To curate comprehensive target information, we extracted the quantitative compound-target interaction profiles from the DTC web-portal (https://drugtargetcommons.fimm.fi/) [34, 35], using the dose-response bioactivity end-points (Kd, Ki and IC_50_). The significance of the kinase targets for each sample was determined using p-values calculated through the permutation test.

### Data analysis and statistics

The DSRT data were processed using the web-based Breeze software [36]. Unsupervised hierarchical clustering of the drug sensitivity profiles was performed and visualised using the Morpheus (https://software.broadinstitute.org/morpheus/). Principal component analysis (PCA) was performed using the ClustVis tool [37]. For synergy assessment, the highest single agent (HSA) synergy model was applied, using the web-application SynergyFinder [38]. Statistical analyses were performed with Prism 9 (GraphPad Software). Correlation plots shown in Fig. 3 and Supplementary Figs. 3A and 9C were made using Spearman’s rank correlation. Limma-voom [39] was used for differential drug sensitivity analysis, based on DSS profiles, and further utilised for drawing the volcano plots. The drugs with DSS ≥ 10 were considered as effective and included in the statistical analysis (Supplementary Fig. 3).

## Results

### Establishment of a functional precision oncology (fPO) pipeline for EOC

**Fig. 1:**
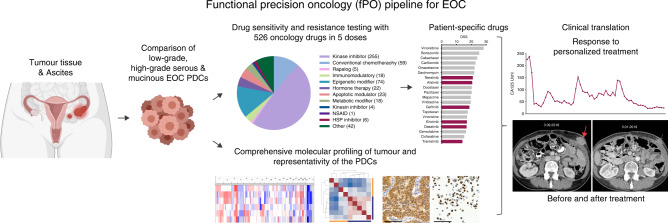
The principle of the functional precision oncology (fPO) pipeline. Patient-derived cancer cells (PDCs) established from HGSOC, LGSOC and MUCOC tumour or ascites samples were subjected to molecular characterisation and functional drug testing platform covering up to 526 drugs to identify patient-selective drug vulnerabilities that can be translated back to the clinics in real-time. Figure was created with BioRender.com.

We developed a fPO approach that uses ascites or fresh ovarian tumour tissue as a starting material from patients with primary or metastatic EOCs for (i) establishment of patient-derived cancer cells (PDCs), (ii) analysis of their genomic and transcriptomic representability, and (iii) integration of genomic, transcriptomic and functional drug sensitivity data (Fig. 1). Five represented LGSOC subtype (FMOC02, FMOC17, FMOC25, FMOC27 and FMOC28), three MUCOC subtype (FMOC03, FMOC06 and FMOC22) and five HGSOC subtype (FMOC04, FMOC09, FMOC11, FMOC14 and FMOC24). Detailed clinical characteristics for each patient case are summarised in the Table 1 and Supplementary Table 1A. Of the HGSOC patient cases, three (FMOC04, FMOC14 and FMOC24) had progressive disease and were resistant to platinum treatment, whereas two other ones (FMOC09 and FMOC11) were sensitive to the neoadjuvant treatment. Four LGSOC and all MUCOC cases were resistant to standard chemotherapy treatments. The PDCs were established from tumour tissue or ascites samples from thirteen patients using culturing protocols that have proven to be effective for development of PDCs for epithelial cancers [18, 21, 40, 41]. We performed genomic and molecular profiling to confirm the representability of each PDC with its original tumour samples (Supplementary Table 1A and Supplementary Fig. 1A–C). PDCs stained positive for pan-cytokeratin, Müllerian marker paired box-protein 8 (PAX8), Tumour protein p53 (TP53) and Wilm’s tumour protein 1 (WT1), which are all known to be commonly expressed in EOC (Supplementary Fig. 1A). Genomic aberrations identified in HGSOC original sample and PDCs included *TP53* mutations, *PIK3CA* copy number gain or amplification, *CCNE1* amplification in three (FMOC04, FMOC09 and FMOC24), *MYC* amplification in one (FMOC11) and *KRAS* amplification in two (FMOC11 and FMOC24) (Supplementary Table S1A and Supplementary Fig. 1B, C). In contrast, all LGSOC PDCs had wild-type *TP53* and less aberrant genomes, with *CDKN2A/B* loss detected in all FMOC2 PDCs, in one out of two FMOC25 PDCs and in OC27 PDC (Supplementary Fig. 1B). For MUCOC, we identified *TP53* mutations (in all three), *KRAS* G12 mutation in FMOC06 and FMOC22, as well as *ERBB2* amplification in FMOC03. Novel fusion genes were identified in FMOC09 (*FNBP4-PTPMT1*) and FMOC11 (*ING5-THAP4* and *ROCK1-SS18*), whereas previously reported oncogenic fusion genes were found in FMOC02 (*CLU-NRG1*), FMOC24 (*RCC1-UBE2D2*) and FMOC25 (*TACSTD2-OMA1*) (Supplementary Table S1G). Fusion genes were further confirmed by RT-PCR and Sanger sequencing (Supplementary Fig. 2A–C). Hierarchical clustering of RNA-sequencing data grouped the PDCs according to EOC subtypes (Supplementary Fig. 2D). LGSOC and MUCOC models exhibited high MAPK pathway gene expression, although no somatic mutations were seen anywhere in this signalling pathway in LGSOC PDCs, whereas MUCOC PDCs FMOC03 had *ERBB2* amplification and both FMOC06 and FMOC22 had KRAS G12 hotspot mutation (Supplementary Fig. 2E).

### Distinct drug efficacies between HGSOC, LGSOC and MUCOC PDCs

**Fig. 2:**
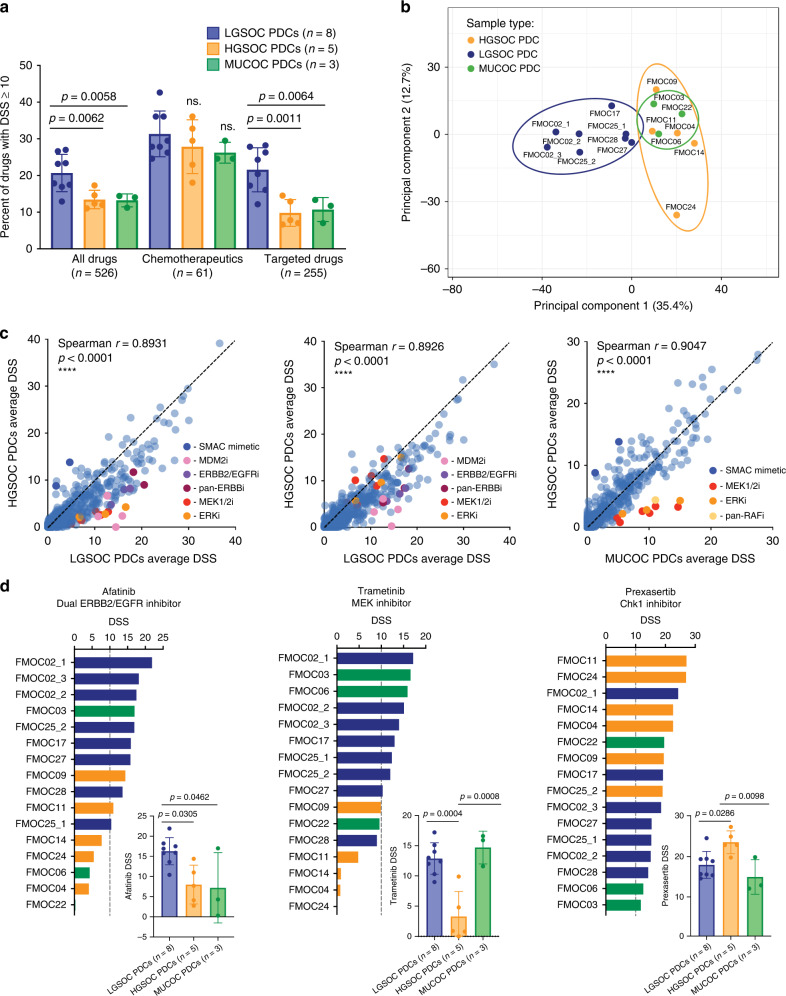
Drug response profiles of EOC PDCs. **a** Percentage of all drugs, chemotherapeutics and targeted drugs with DSS ≥ 10 across LGSOC, HGSOC and MUCOC PDCs. Data are presented as means ± SEMs. *p* value from two-tailed Welch’s *t* test is shown. **b** Clustering of the overall drug response profiles across EOC PDCs to the panel of up to 526 approved and investigational oncology compounds by principal component analysis (PCA). **c** Pairwise correlation between the average DSS levels of LGSOC and HGSOC, LGSOC and MUCOC, and HGSOC and MUCOC PDCs. Drug families showing highest differences between compared EOC subtypes are highlighted in colours in each subplot. **d** The response to the irreversible dual ERBB2/EGFR inhibitor afatinib, MEK inhibitor trametinib and Chk1 inhibitor prexasertib across all the EOC PDCs presented as waterfall plots and comparisons of average drug efficacies between the EOC subtypes. Data are presented as means ± SEMs. *p* values from one-way ANOVA test.

We carried out drug sensitivity testing of sixteen PDCs with a panel of up to 526 approved drugs and investigational oncology compounds (Supplementary Tables S1H and S1I). The drug testing assay was reproducible as validated by the replicate screens (Supplementary Fig. 3A). The same drug library has been used in several other studies to identify novel drug vulnerabilities in leukaemias and solid tumour PDCs and established cell lines [14, 22, 42–44]. The library includes among other drug classes 255 small-molecule kinase inhibitors covering both receptor tyrosine (e.g., ERBB2 and EGFR) and non-receptor tyrosine (e.g., PI3K/mTOR) kinase inhibitors. We defined a drug moderately to strongly effective if its drug sensitivity score (DSS) value exceeded the 85% quantile (DSS ≥ 10) of the overall DSS distribution (Supplementary Fig. 3B). Of the 526 tested compounds, 20.7% had DSS ≥ 10 in LGSOC (on average 109 drugs), 13.5% in HGSOC (on average 71 drugs) and 13.2% in MUCOC PDCs (on average 70 drugs), implying that LGSOC PDCs were overall more sensitive to all drug classes (Fig. 2a). This significant sensitivity difference was due to responses to the targeted drugs (21.6% had DSS ≥ 10 in LGSOC, 9.8% in HGSOC and 10.7% in MUCOC PDCs), whereas there was a modest change in sensitivity to chemotherapeutics among the three types of EOC PDCs (31.4% in LGSOC, 27.9% in HGSOC and 26,2% in MUCOC PDCs) (Fig. 2a). The variability in the drug response profiles was further visualised by principal component analysis (PCA), which showed that LGSOC PDCs formed their own cluster, separate from the HGSOCs and MUCOCs (Fig. 2b).

Average DSSs in HGSOCs and LGSOC models showed higher sensitivities to drugs targeting MDM2, ERBB2/EGFR and MEK/ERK, whereas comparison of the average DSSs between HGSOC and MUCOC PDCs showed higher sensitivity to MEK/ERK inhibitors (Fig. 2c). For example, afatinib, a dual irreversible ERBB2/EGFR inhibitor showed significantly higher efficacy in LGSOC PDCs (DSS ≥ 10), compared to HGSOC (*p* = 0.0305) and MUCOC (*p* = 0.0462) (Fig. 2d). The potential importance of ERBB2 and EGFR signalling in the LGSOC was further supported by the target addiction scoring (TAS), which makes use of multi-target drug polypharmacology to map the complex interconnections between drug responses and their target interaction profiles (Supplementary Fig. 4). TAS integrates both primary protein targets as well as potent off-targets of drugs to provide a ranking of potential therapeutic targets according to their functional importance in each cancer sample [32, 33]. MEK inhibitor trametinib showed higher sensitivity in LGSOC (*p* = 0.0004) and MUCOC PDCs (*p* = 0.0008), when compared to HGSOC PDC (Fig. 2d). Chk1 inhibitor prexasertib showed higher sensitivity in HGSOC, compared to LGSOC (*p* = 0.0286) and MUCOC (*p* = 0.0098). This compound is undergoing a phase 2 clinical trial (NCT03414047) in HGSOC lacking BRCA1/2 mutations (Fig. 2d).

We identified potential actionable compounds that are either clinically approved for patient treatment or are undergoing clinical trial for each subset of EOCs, using selective DSS, where the drug responses of the PDCs were compared with the average responses of healthy bone marrow derived mononuclear cells to exclude drugs that showed cytotoxicity in the healthy cells (Table 1 and Supplementary Figs. 5 and 6). For example, Wee1 inhibitor AZD1775 and Chk1 inhibitor prexasertib were among the top selective drugs for the FMOC04 PDC, which harbours *CCNE1* amplification. It has been shown that combination of AZD1775 and gemcitabine improves the progression-free survival in platinum-resistant recurrent HGSOC patients in phase 2 clinical trial [45].

### Selective responses of LGSOC PDCs to MDM2 inhibitors

**Fig. 3:**
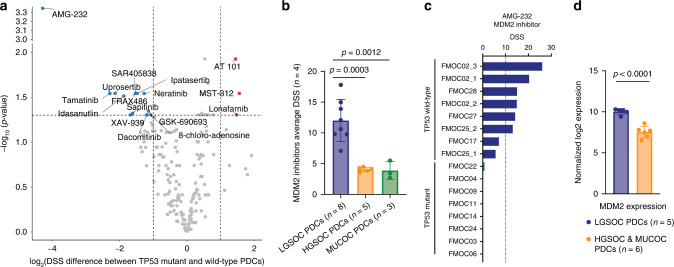
Selective responses of LGSOC PDCs to MDM2 inhibitors. **a** Volcano plot of differences in drug sensitivity across EOC PDCs with *TP53* wild-type (all LGSOC PDCs) and mutant *TP53* (all HGSOC and MUCOC PDCs). Highlighted drugs show selective sensitivity towards *TP53* wild-type (in blue) or mutated (in red) EOC PDCs. Drugs with DSS ≥ 10 were included in the Volcano plot. **b** The average responses to MDM2 inhibitors (*n* = 4) across EOC PDCs. Data are presented as means ± SEMs. *p* values from one-way ANOVA. **c** The responses to MDM2 inhibitor AMG-232 across EOC PDCs. D) RNA-seq CPM-based normalised expression of MDM2 in 5 LGSOC PDCs and 6 HGSOC and MUCOC PDCs. Data are presented as means ± SEMs. *p* value from two-tailed Welch’s *t* test.

We also observed higher sensitivity of MDM2 inhibitors in LGSOC PDCs that have wild-type *TP53* in comparison to HGSOC and MUCOC PDCs with mutant *TP53* (Fig. 3a). Notably, MDM2 inhibitors AMG-232, idasanutlin and SAR405838 exhibited strong selective responses in LGSOC PDCs (*p* = 0.0003, LGSOC PDCs vs. HGSOC PDCs, Fig. 3b). AMG-232 has shown encouraging results in phase 1 study in *TP53* wild-type solid tumours [46], and it had the highest sensitivity in LGSOC PDCs among all MDM2 inhibitors (Fig. 3c). The analysis of *MDM2* mRNA expression by RNA-seq across all PDCs revealed significantly higher MDM2 levels in LGSOC PDCs, when compared to other samples with mutant *TP53* (*p* < 0.0001, Fig. 3d). Among the other targeted drugs displaying LGSOC-specific responses were dual ERBB2/EGFR inhibitor neratinib, AKT inhibitor ipatasertib and Syk inhibitor tamatinib (Fig. 3a).

### SHP2 phosphatase inhibitor SHP099 synergises with MEK inhibitors in PDCs

**Fig. 4:**
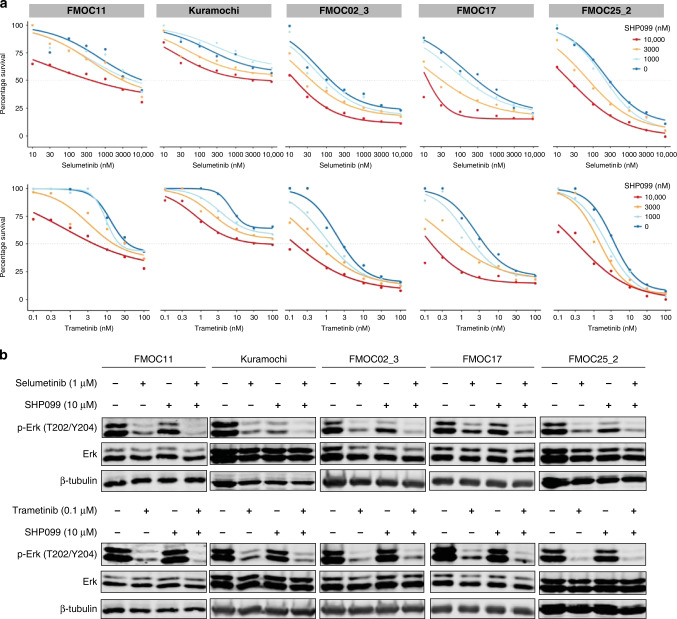
Combined SHP2 and MEK inhibition synergistically inhibits EOC PDCs growth. **a** Dose-response curves of selumetinib and trametinib alone and in combination with SHP099 (1000 nM, 3000 nM and 10,000 nM) in PDCs (FMOC11, FMOC02_3, FMOC17 and FMOC25_2) and Kuramochi cell line measured with cell viability assay after 1 week. Shown is one representative experiment of at least three replicates. The individual 2D synergy plots for each sample are presented in Supplementary Fig. 7. **b** Immunoblots for phospho-ERK and total ERK levels in lysates of PDCs and Kuramochi cell line treated with DMSO, selumetinib, trametinib, SHP099 or combinations as indicated. β-tubulin was used as a loading control.

Recently, Fedele et al. showed that protein-tyrosine phosphatase (SHP2) inhibitor SHP099 in combination with MEK inhibitor (either trametinib or selumetinib) blocked proliferation of multiple cancer cell lines, including OC, and prevented the development of adaptive resistance to MEK inhibitors [47]. To explore whether this combination is also effective in EOC PDCs, we tested its potency in FMOC11 (HGSOC with amplification and overexpression of wild-type *KRAS*) and in three LGSOC models (FMOC02_3, FMOC17 and FMOC25_2), which all harbour wild-type *KRAS* but show elevated expression of RAS-pathway components by RNA-sequencing data (Supplementary Fig. 2E). Potential synergistic interactions were tested using 7 × 7 multi-dose combination matrices and the SynergyFinder was used to score any potential synergy [38]. SHP099 alone had almost no effect on cell viability, whereas MEK inhibitors selumetinib and trametinib had moderate single-agent efficacy (Fig. 4a and Supplementary Fig. 7). The combination of SHP099 with either selumetinib or trametinib showed synergy in all four tested PDC models and in a control, Kuramochi cell line, that has KRAS amplification (Fig. 4a and Supplementary Fig. 7). The combinations of both SHP099 and selumetinib (24 h treatment) and SHP099 and trametinib (48 h treatment) inhibited ERK phosphorylation in all the tested PDCs (Fig. 4b). Furthermore, to examine the long-term combined effects of SHP2 and MEK inhibition on EOC PDCs, we performed colony formation assay, where cells were exposed to SHP099 (10 µM), trametinib (1–10 nM) and selumetinib (0.1–1 µM), either individually or in combination for up to two weeks. The results showed that the combination treatment of SHP099 with either trametinib or selumetinib led to substantially enhanced inhibition in all the models, thus corroborating our initial drug combination testing results (Supplementary Fig. 8A, B). These results support the combinatorial inhibition of SHP2 and MEK as a potential therapeutic strategy for EOCs with activated MAPK pathway, and further illustrate the value of ex vivo PDCs in testing combinations of existing and emerging drugs.

### Clinical application of fPO in an LGSOC patient with a *CLU-NRG1* gene fusion activating ERBB3 signalling

**Fig. 5:**
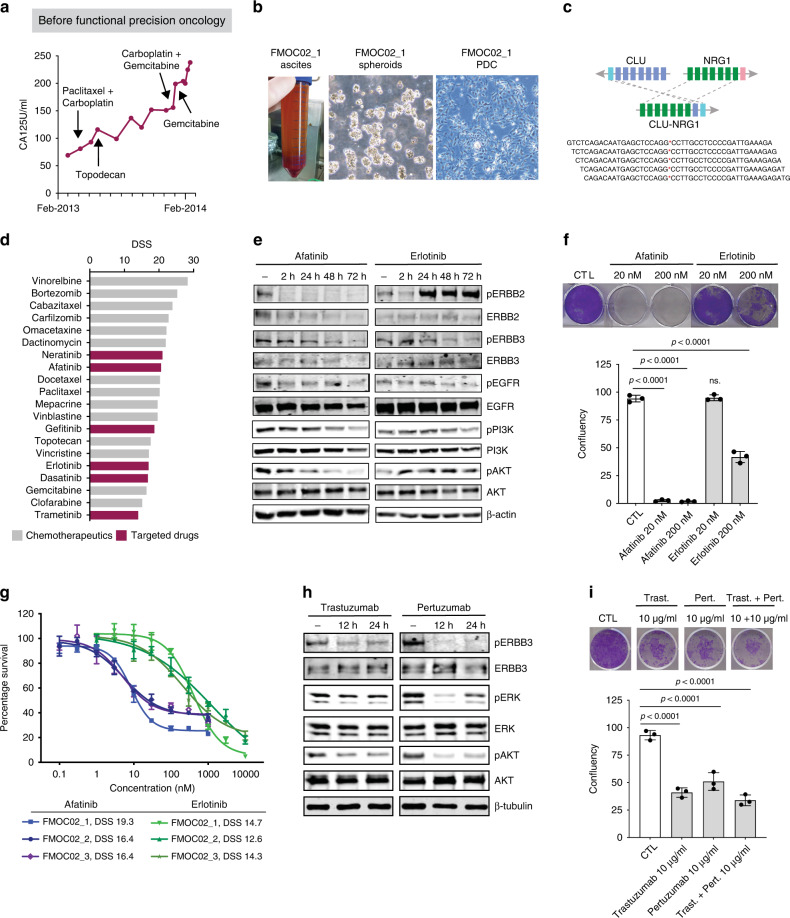
*CLU-NRG1* fusion gene is targetable by anti ERBB2/ERBB3 pharmacological agents. **a** Schematic representation of FMOC02 patient’s CA125 levels, measured after relapse during standard chemotherapy treatments. **b** Picture of the original FMOC02_1 ascites sample, the tumour spheroids and established FMOC02_1 PDC. Scale bar, 100 µM. **c** Schematic representation of *CLU-NRG1* rearrangement showing genomic structure of the fusion gene and some of the fusion-point spanning sequences. **d** Waterfall plot showing the most effective approved chemotherapy (grey) and targeted (purple) drugs for FMOC02_1 PDC based on drug sensitivity score (DSS). **e** Phosphorylation of ERBB receptors and downstream signalling transducers of FMOC02_1 PDC in dose-response experiments. FMOC02_1 PDCs were treated with afatinib or erlotinib (200 nM) for different time points as indicated, followed by cell lysing and immunoblotting with indicated antibodies. β-actin was included as a loading control. **f** Long-term CFA with FMOC02_1 PDCs cultured for 14 days in the absence or presence of afatinib and erlotinib (20 or 200 nM). Colonies were counted with Image J using the ColonyArea plugin and shown as percentage inhibition by comparison with untreated cells. Shown results are representative of at least two independent experiments. Data are presented as means ± SEMs. *p* values from one-way ANOVA is shown. **g** Dose-response curves of afatinib and erlotinib for the three FMOC02 PDCs. **h** Phosphorylation of ERBB3, ERK and AKT in FMOC02_3 PDCs treated with trastuzumab and pertuzumab for 12 and 24 h (10 μg/ml), followed by protein extraction and immunoblotting. β-tubulin was included as a loading control. **i** Long-term CFA with FMOC02_3 PDCs cultured for 14 days in the absence or presence of trastuzumab (10 μg/ml), pertuzumab (10 μg/ml) and their combination (both 10 μg/ml). Colonies were counted with Image J using the ColonyArea plugin and shown as percentage inhibition by comparison with untreated. Shown results are representative of at least two independent experiments. Data are presented as means ± SEMs. *p* values from one-way ANOVA.

**Fig. 6:**
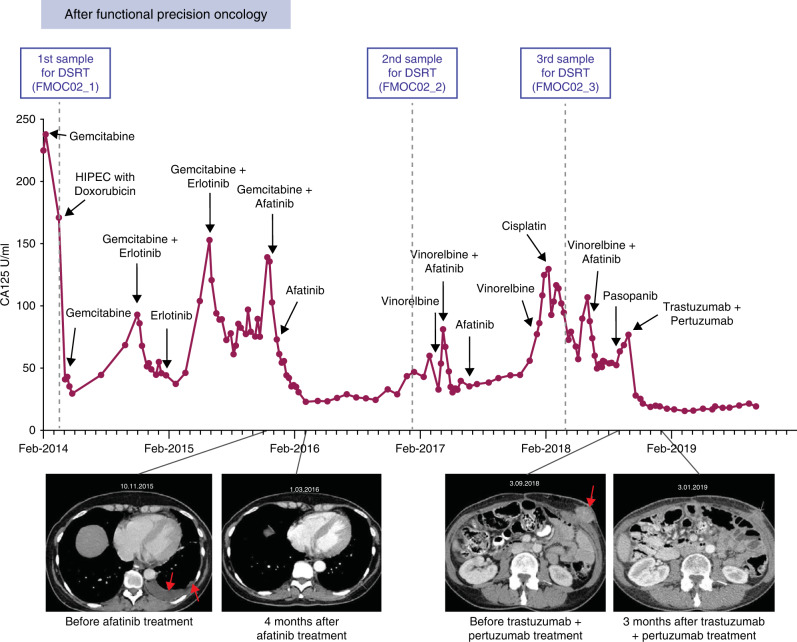
Functional precision oncology-guided treatment for FMOC02 in the clinic. Schematic representation of FMOC02 patient’s CA125 levels, measured during different treatments guided by the functional precision oncology approach. The dots indicate CA125 measurement timepoints, black arrows mark the start of a new clinical treatment, minor ticks on the x-axis refer to the months and major ticks to the years. Bottom: representative CT scans, linked with the time-point of the treatment. Red arrows point toward the tumour in the CT scan.

One of the LGSOC patients (FMOC02) experienced a relapse 17 years after diagnosis and primary operation. Standard chemotherapy had no effect on cancer progression as evidenced by the continuously rising CA125 serum levels (Fig. 5a). A HIPEC (hyperthermic intraperitoneal chemotherapy) with doxorubicin was performed in 2014 and PDC FMOC02_1 was established from the ascites sample (Fig. 5b). DNA sequencing revealed a loss of the *CDKN2A* locus at chromosome 9 and no other driver mutations, while RNA-sequencing detected a *CLU-NRG1* fusion gene, which resulted in a rearrangement of exons 1 to 2 of clusterin (*CLU*) gene with exons 2 to 13 of the neuregulin-1 (*NRG1*) gene (Fig. 5c and Supplementary Fig. 2B, C). Therefore, the EGF-like domain of the *NRG-1* was driven by the *CLU* promoter. The NRG1 ligand induces the heterodimerization and activation of ERBB2/ERBB3 signalling [48]. Accordingly, we detected a strong phospho-ERBB2 and phospho-ERBB3 expression in FMOC02_1 PDC and in the original peritoneal metastasis sample by IHC (Supplementary Fig. 9A). Moreover, drug testing of FMOC02_1 PDC identified high sensitivity to afatinib/neratinib (second-generation irreversible dual EGFR/ERBB2 inhibitors) and to erlotinib/gefitinib (first-generation reversible EGFR inhibitors) (Fig. 5d). We further validated this finding in 3D culture condition (Supplementary Fig. 9B). Based on the fPO results, the patient was first treated with a combination of gemcitabine and erlotinib resulting in a favourable response (Fig. 6). When CA125 level started to increase after 12 months of treatment, the patient received afatinib, first in combination with gemcitabine followed by afatinib monotherapy, resulting in a marked decrease in CA125 levels (Fig. 6). We also confirmed better efficacy of afatinib over erlotinib in inhibiting phospho-AKT levels or colony formation in ex vivo FMOC02_1 PDC (Fig. 5e, f).

When CA125 levels increased again 12 months later, a needle biopsy and ascites samples were obtained and used to generate FMOC02_2 in 2017 and FMOC02_3 in 2018, respectively. The presence of *CLU-NRG1* fusion and the loss of *CDKN2A* was detected in both samples (Supplementary Figs. 1B and 2B). The drug response profiles observed in all three FMOC02 PDCs were remarkably similar and showed continued dependency of cancer cells on the ERBB2/EGFR and RAS/ERK signalling (Fig. 5g and Supplementary Figs. 6 and 9B–D). We therefore next tested whether anti-ERBB2/ERBB3 monoclonal antibodies trastuzumab and pertuzumab had any effect on FMOC02 PDCs. Treatment with either trastuzumab or pertuzumab showed decreased phospho-AKT and phospho-ERK levels and inhibition of colony formation, demonstrating the efficacy of these antibodies in blocking NRG1/ERBB3 activation and its downstream signalling (Fig. 5h, i). Subsequently, the patient received trastuzumab and pertuzumab in combinatorial therapy, which resulted in stabilised CA125 levels and disappearance of metastases in the CT (Fig. 6). Notably, all three FMOC02 PDCs established over a 4-year period showed excellent responses to ERBB inhibitors and the presence of the same key genomic events and molecular drivers.

## Discussion

Here, we demonstrate how the fPO data from the functional profiling of EOC PDCs cells provides actionable information about the dependency of the cancer on key signalling pathways, and on vulnerability or resistance to drugs or drug combinations. While the efficacy of targeted agents can sometimes be predicted from cancer genomics data alone, synthetic lethalities, non-genomic dependencies, and other complex vulnerabilities to drugs are much more difficult to predict from genomic profiles alone. This is also the case for drug combinations. The concept of matching drugs to patients based on functional testing of patient-derived cells ex vivo is already being tested in clinical trials in both haematological malignancies and solid tumours [49]. In solid tumours, it is more challenging to develop and ascertain representativity of ex vivo models for functional precision oncology purposes, with the aim to provide actionable insights for patient treatment.

Applying PDCs for guiding functional precision medicine studies in different types of solid tumours has been proposed as a new strategy for precision medicine, but very little clinical evidence exists so far [18, 19, 41]. For example, organoid models are intensively investigated as advanced models, but their suitability for real-time translational application and for high-throughput drug screening purposes is limited by the time needed to establish and expand such models [12, 13].

Previous studies have shown that the genomic and molecular profiles of LGSOCs, MUCOCs and HGSOCs are quite distinct in terms of e.g. *TP53*, *KRAS* and *BRAF* mutation status [5]. Therefore, our aim was to investigate the overall drug response profiles of representative type I (LGSOC and MUCOC) and type II (HGSOC) EOC PDCs to a library of 526 drugs and to identify both subtype-specific and patient-specific drug vulnerabilities. Our results indicated that while each PDC was unique, the three EOC subgroups had a distinct overall drug response profile. LGSOC PDCs exhibited on average strong responses to inhibitors of *ERBB*-gene family, MDM2, MEK, and PI3K/mTOR as well as to the multi-kinase inhibitor dasatinib, MUCOC PDCs were sensitive to MEK and ERK inhibitors, while all HGSOC PDCs were sensitive to PI3K/mTOR inhibitors.

Since drug response profiling revealed that HGSOC exhibit increased resistance to targeted drugs, we sought to identify possible combination therapies that could effectively kill HGSOC cancer cells. For this purpose, we focused on drugs targeting MAPK pathway that play an important role in EOC progression [50]. According to the TCGA data, 11% of HGSOC cases have *KRAS* amplification [50]. Mutations in either KRAS, BRAF or other components of MAPK pathway are also common in LGSOC [51], and in MUCOC KRAS mutations are found in up to 40–65% cases [52]. Recent studies have demonstrated a strong synergy between SHP2 kinase inhibitor SHP099 and MEK inhibitors [47, 53]. Consistent with these observations, combination treatment of SHP099 with either trametinib or selumetinib had a better inhibitory effect in all of our tested PDCs models, both in short-term testing and in long-term colony forming assays. This included the HGSOC patient case FMOC11 with *KRAS* amplification, indicating that simultaneous targeting of SHP2 and MEK signalling pathway could improve MEK inhibitor efficacy in RAS-mutant EOC. In addition, we found similar effects in EOC cases with high expression of wild-type RAS, suggesting that the effects of MEK inhibition by itself and the joint effect of MEK and SHP2 inhibition may not be limited to RAS mutant cancers.

We were able to establish representative EOC PDCs in a clinically actionable time-frame for all cases, while translation to clinical treatment was possible only in one case. We describe a LGSOC case with an oncogenic fusion gene *CLU-NRG1* (FMOC02), where fPO was applied to tailor treatment for a patient who was unresponsive to a conventional chemotherapy. NRG1 is the ligand for ERBB/EGFR receptors, and NRG1/ERBB3 activation loop has been linked to EOC progression [48]. Moreover, *NRG1*-fusion genes are potentially actionable genomic events in other cancers such as lung cancer [54]. *CLU-NRG1* has been detected in EOC, such as in HGSOC [55]. Based on functional drug responses of FMOC02 PDCs, the patient was initially treated with a combination of erlotinib and gemcitabine, followed by EGFR/ERBB2 dual inhibitor afatinib, and ultimately with a combination of anti-ERBB2 monoclonal antibodies trastuzumab and pertuzumab. The initiation of each of these multiple therapies led to positive responses and eventually kept the disease under control for over 5 years. Therefore, this is an example of how functional precision medicine could be applied in the future to tailor patient treatments. A few aspects make LGSOCs a particularly attractive model for implementing functional precision oncology in solid tumours. First, LGSOC is often metastatic, but responds poorly to conventional chemotherapies. Thus, functional precision medicine can illuminate targeted therapies that may prove effective. Second, LGSOCs, like our FMOC02 patient case, often show wild-type *TP53* and low level of genomic instability, and hence resistance to targeted treatments is not as common as in other tumour types. Third, ERBB inhibition with erlotinib and pertuzumab was shown to result in clinical responses in two patients with *NRG1*-rearranged pancreatic ductal adenocarcinoma (PDAC) tumours, which is in line with our results and suggesting therapeutic options for tumours with aberrant ERBB receptor-mediated signalling arising from NRG1 activation [56]. Our findings support the use of ERBB-targeted inhibitors for *NRG1*-rearranged EOC, although this will need to be proven in formal clinical trials.

In conclusion, drug response profiling with genotypically and phenotypically representative PDCs can be utilised to identify and validate cancer driver signals and to pinpoint clinically actionable inhibitors and their combinations that could be applied for real-time patient treatment optimisation and personalisation. We acknowledge some of the limitations inherent in all ex-vivo culture technologies, such as the lack of a complex tumour microenvironment or immune cells. These could in the future be addressed with e.g. complex models and explant tissue cultures, which on the other hand, are inherently low-throughput technologies. Functional precision medicine is expected to significantly expand the actionability of the current, mostly genomics-based personalised medicine approaches, and help in drug repositioning to new indications. In this study, we have demonstrated the proof-of-concept implementation of EOC PDCs in guiding treatment decisions for LGSOC, highlighting promising drug efficacies along the ERBB family inhibition that may have clinical significance.

### Supplementary information

Supplementary Figures

Supplementary Tables

## Data Availability

The data generated during the study are presented in the main and supplemental figures and included in the supplemental materials. Patient-derived data are protected and are not broadly available due to privacy laws, ethical considerations and patient consent. Anonymised data may be requested from the corresponding authors with appropriate institutional approvals.
