# Supplementary material for: Drug response profiles in patient-derived cancer cells across histological subtypes of ovarian cancer: real-time therapy tailoring for a patient with low-grade serous carcinoma
Source: Br J Cancer. 2022 Dec 7;128(4):678–90. doi: 10.1038/s41416-022-02067-z (PMC9938120; doi:10.1038/s41416-022-02067-z)
Supplement: Supplementary file 1 — Supplementary Figures [file 41416_2022_2067_MOESM1_ESM.pdf]

Supplementary Figure 1

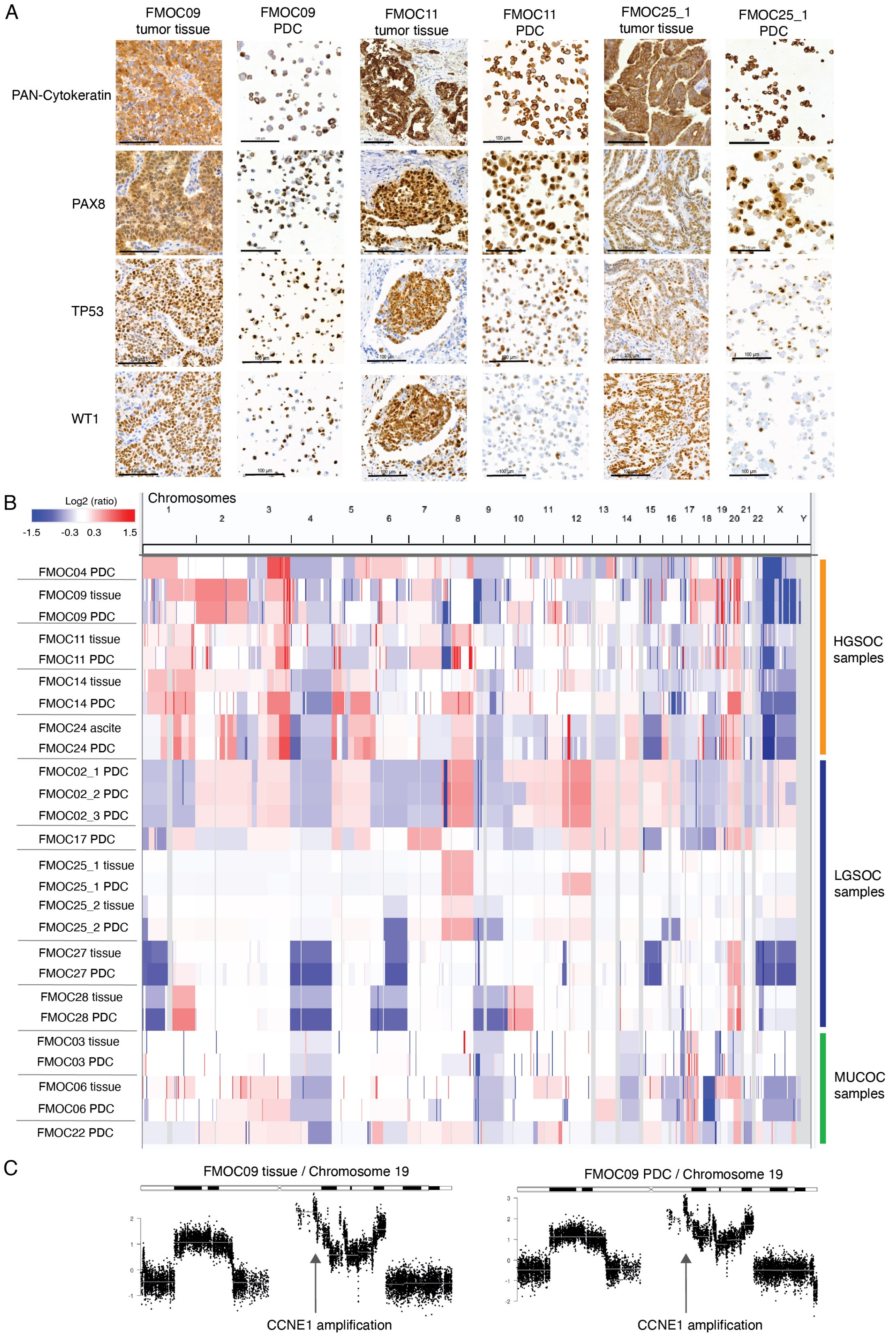

Supplementary Figure 1. Genomic and phenotypic profiling of EOC PDC models. A) Representative immunohistochemistry (IHC) images of the formalin-fixed paraffin-embedded (FFPE) sections from FMOC09, FMOC11 and FMOC25_1 original tumor tissues and PDCs stained with pan-cytokeratin, PAX8, TP53 and WT1. Scale bar, 100 μm. B) Copy number variation (CNV) profiles for HGSOC, LGSOC and MUCOC PDC models and original tumor samples. C) Overlapping CNV profiles of FMOC09 original tissue and PDC chromosome 19. High level *CCNE1* amplification is indicated with an arrow in both samples. CNV profiles are plotted against genomic coordinates on horizontal axis for each sample.

Supplementary Figure 2

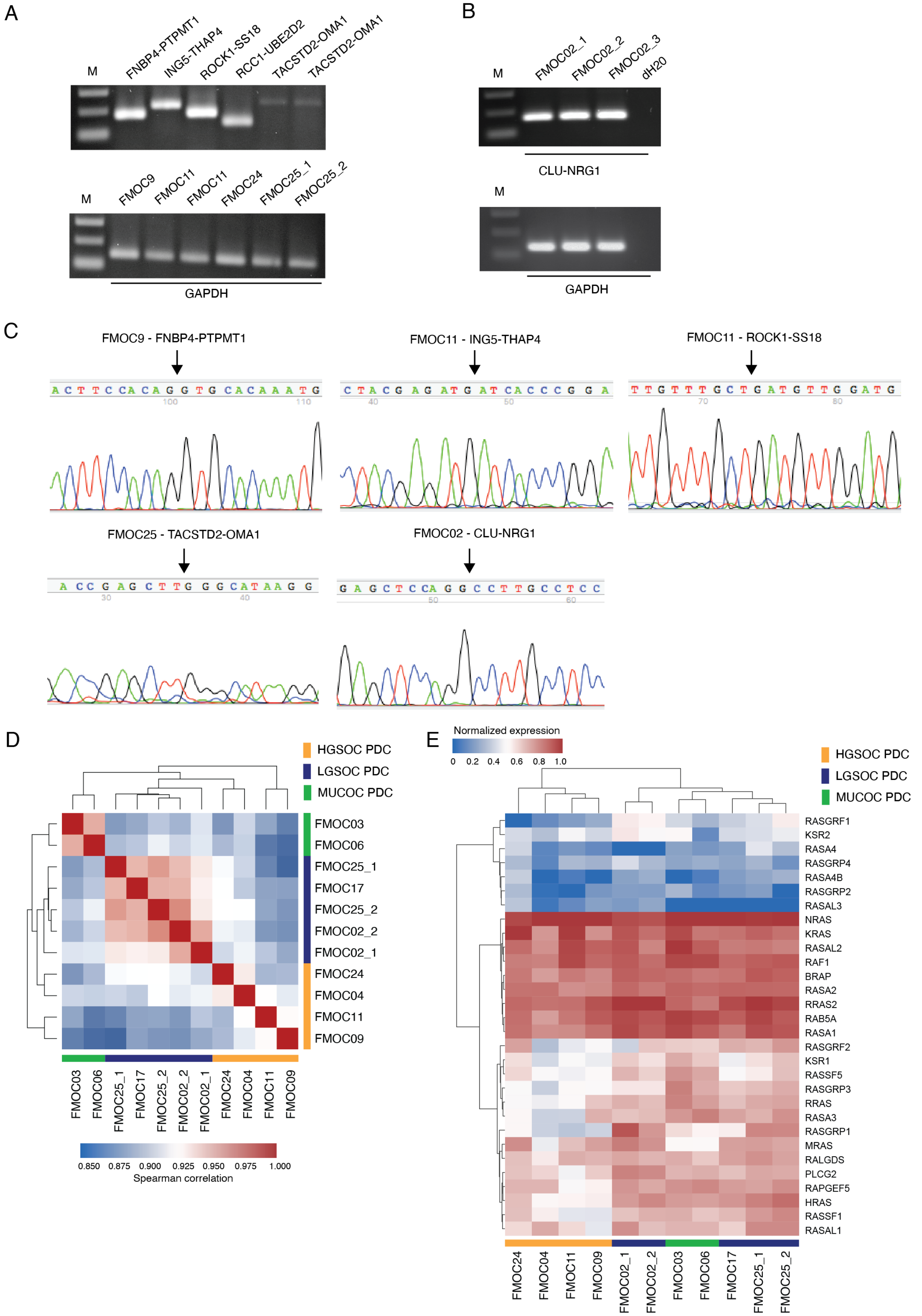

Supplementary Figure 2. Transcriptomic profiling of EOC PDC models. A) RT-PCR validation of fusion genes in FMOC09, FMOC11, FMOC24 and FMOC25 PDCs. B) RT-PCR validation of *CLU-NRG1* fusion gene in FMOC02 PDCs FMOC02_1, FMOC02_2 and FMOC02_3. RT-PCR, reverse transcriptase PCR. C) Sanger sequencing validation of fusion genes. Fusion breakpoint is indicated with an arrow. D) Spearman´s rank correlation for the similarities between EOC PDCs gene expression data. E) Hierarchical clustering of EOC PDCs by expression of Ras-pathway genes. Counts per million (CPM) values were log2 transformed and normalized by sample (column) to scaled values (0–1), distances calculated with the Euclidean distance metric and clustering determined by the Ward variance minimization algorithm.

Supplementary Figure 3

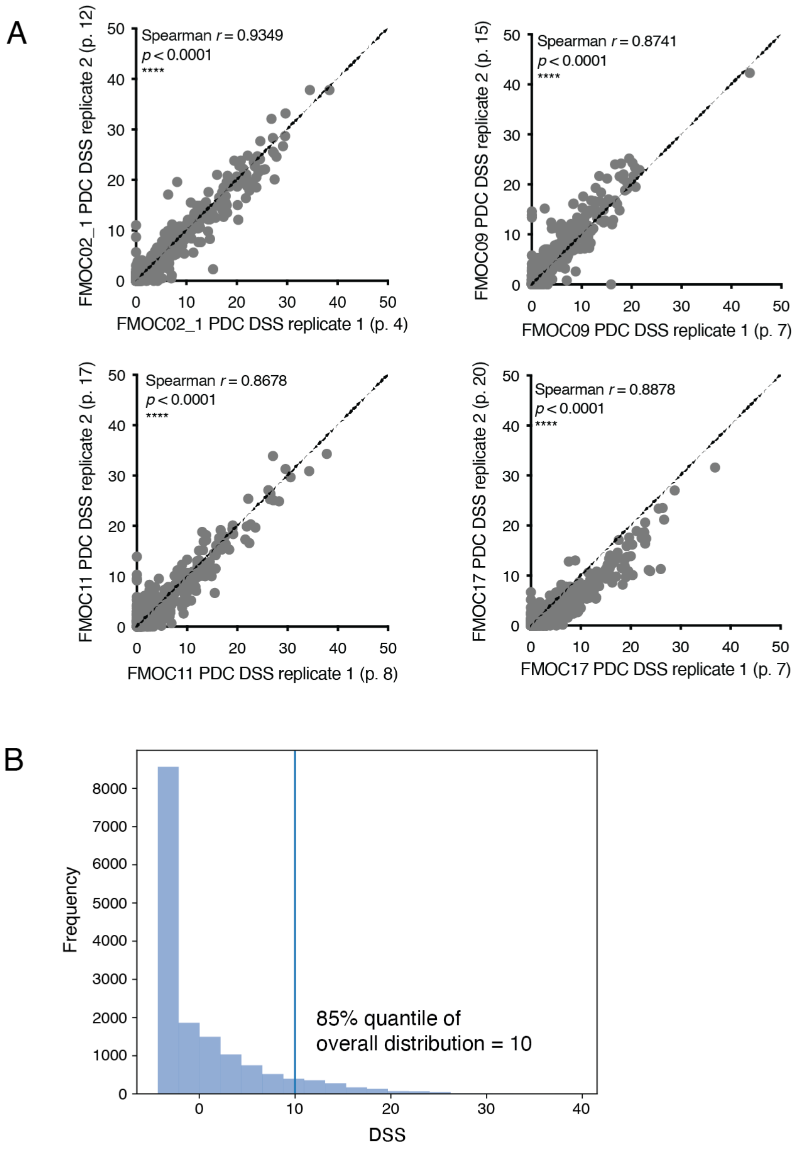

Supplementary Figure 3. A) Comparison of DSRT results from the replicate screens with four EOC PDCs. The passage number for each PDCs in shown in the parenthesis. B) Overall distribution of DSS values across OC PDCs showing that DSS value 10 corresponds to 85% quantile of the distribution.

Supplementary Figure 4

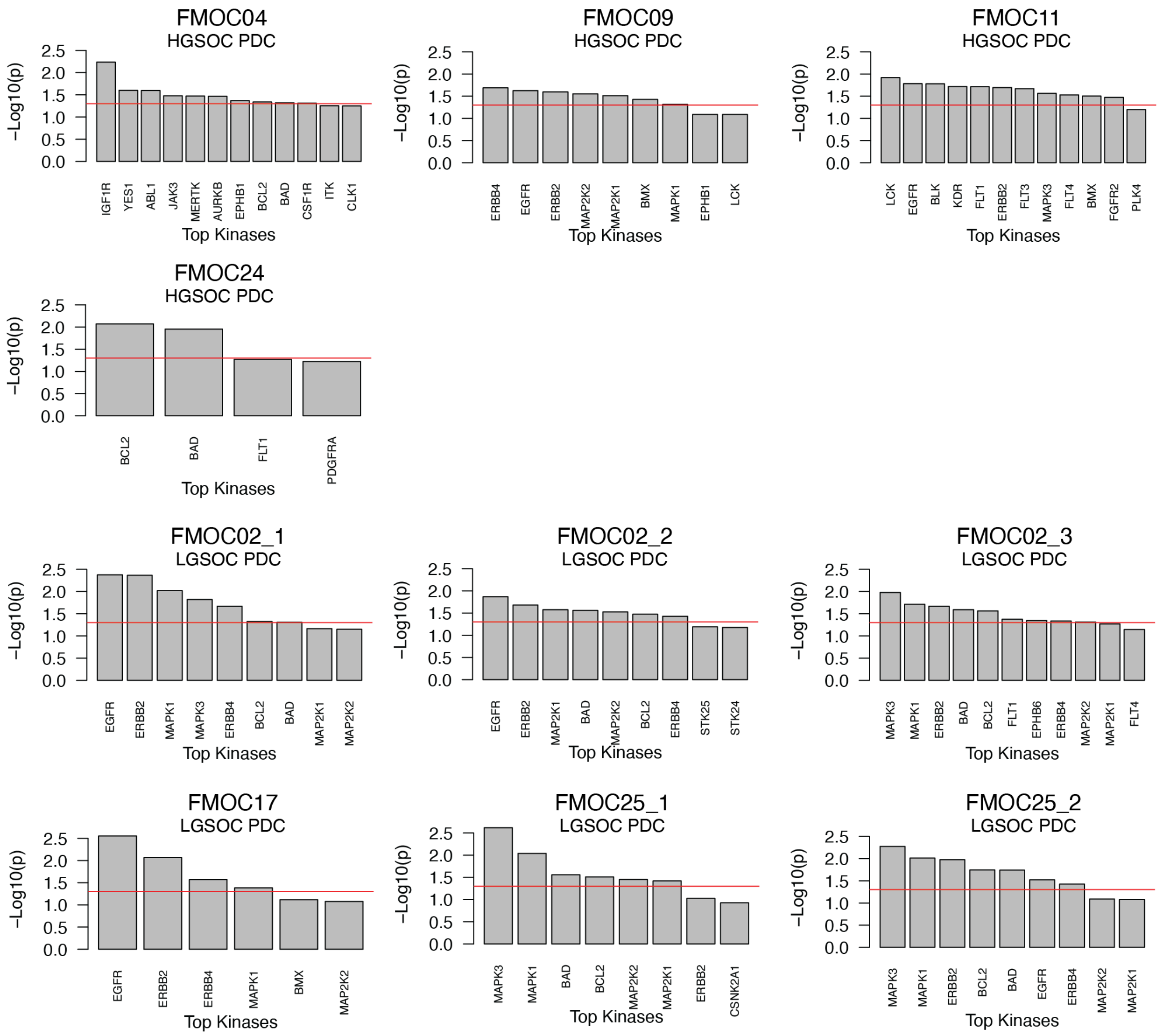

Supplementary Figure 4. Target addiction scoring (TAS) analysis across EOC PDCs based on drug responses and target information predicts the functional importance of individual kinases for inhibiting each PDC. Red horizontal line indicates the significance cut-off of p=0.05 based on permutation testing.

Supplementary Figure 5

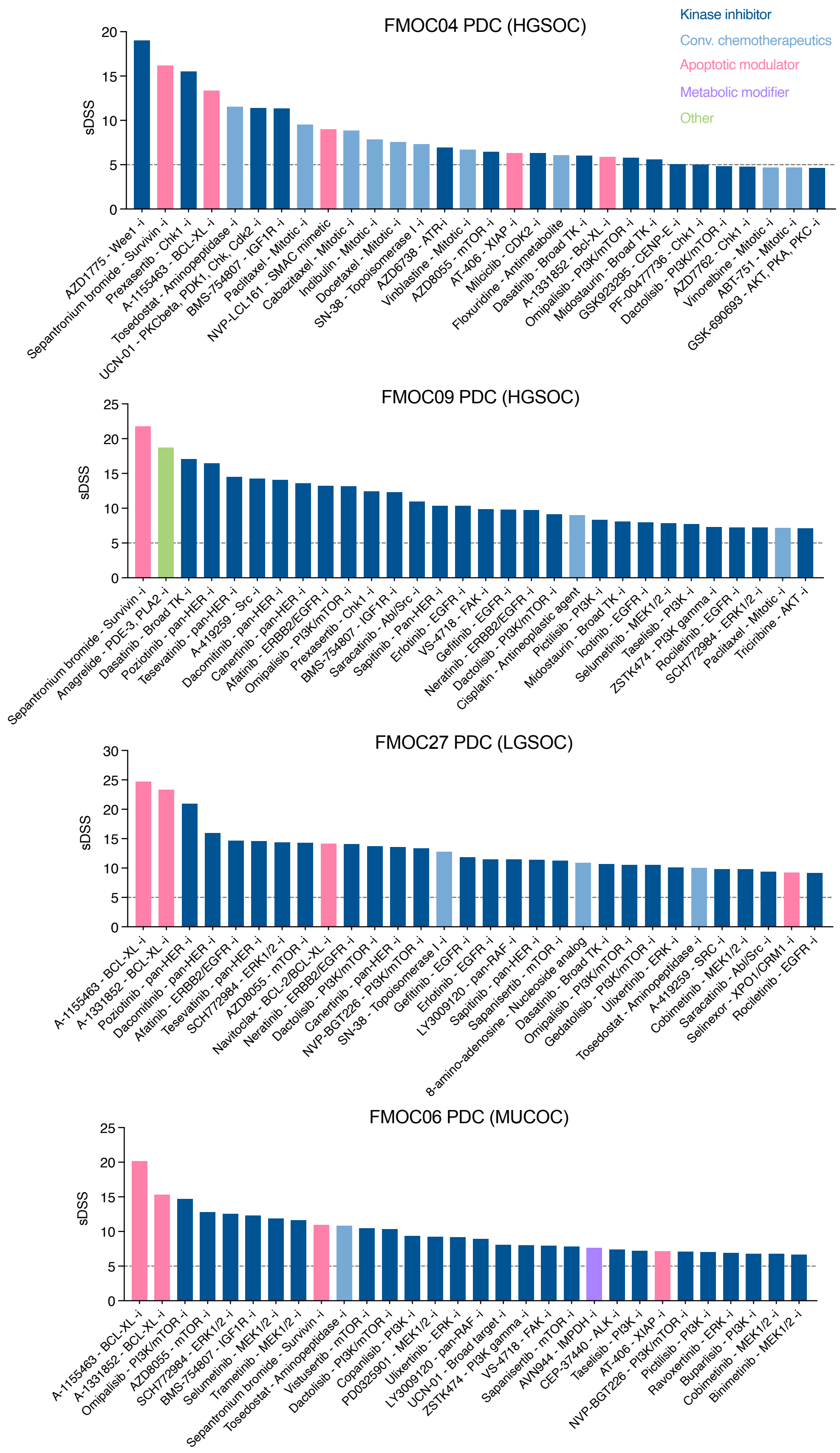

Supplementary Figure 5. Thirty most effective drugs for selected EOC PDCs based on selective DSS. The identity of each PDC and EOC subtype is presented on top of the individual plots.

Supplementary Figure 6

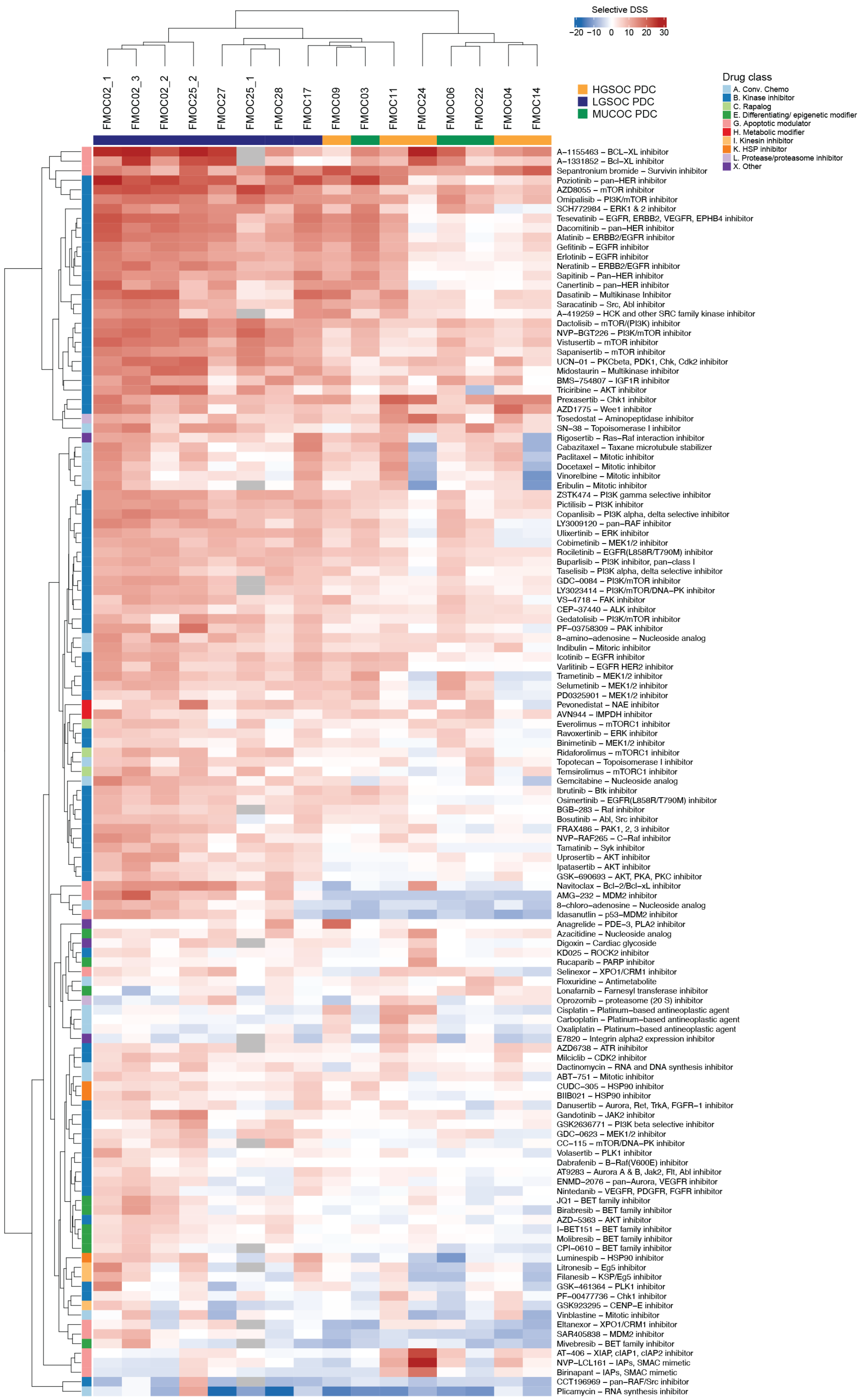

Supplementary Figure 6. Drug response heatmap for EOC PDCs based on selective DSS. Unsupervised clustering of overlapping drugs with a DSS ≥ 10, in at least one sample. Different drug classes are shown in distinctive colors next to the heatmap. Columns represent samples and rows represent drugs. Red indicates higher DSS whereas blue indicates lower DSS in comparison to the average of two healthy bone marrow samples (selective DSS).

Supplementary Figure 7

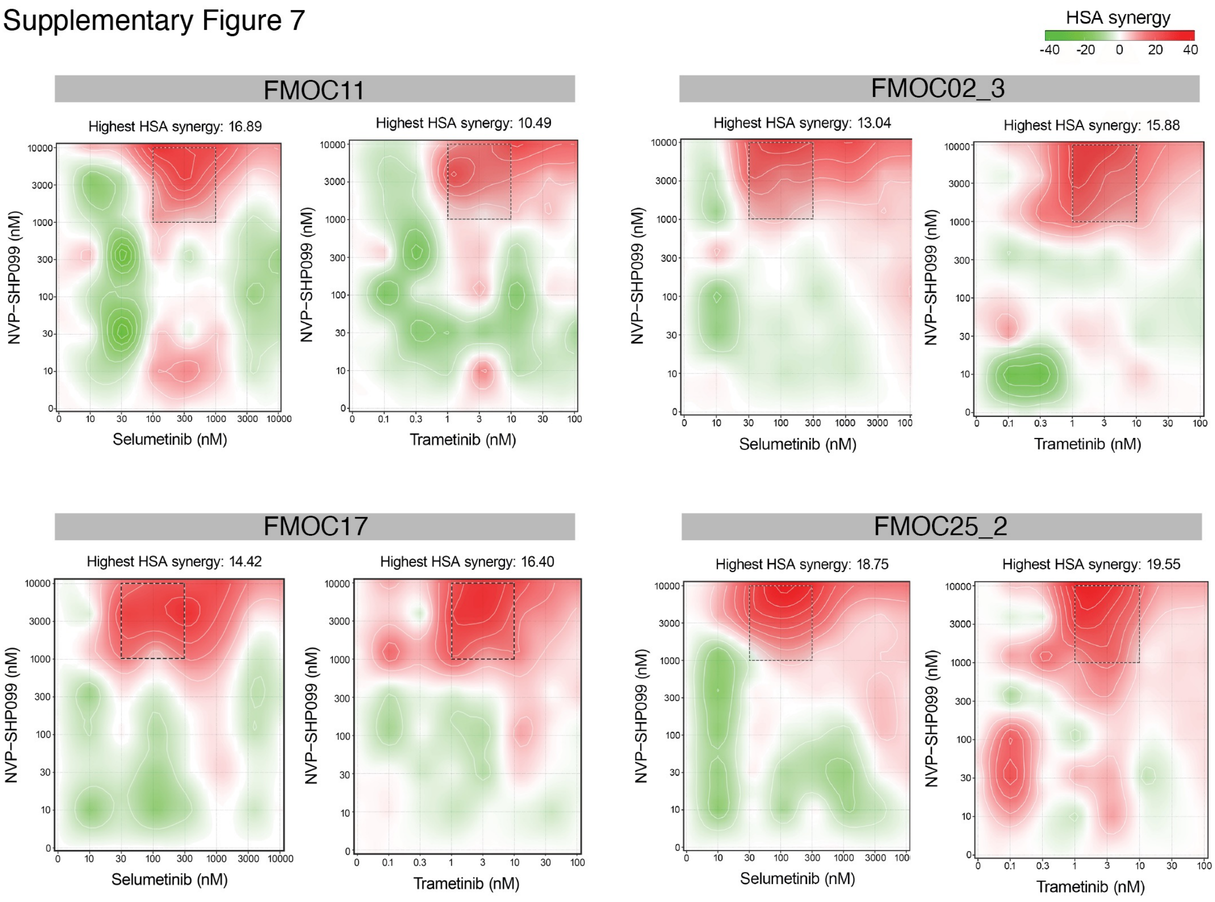

Supplementary Figure 7. SHP2 inhibitor SHP099 in combination with MEK inhibitors selumetinib and trametinib synergistically inhibits EOC PDCs cell growth. EOC PDCs (FMOC11, FMOC02_3, FMOC17 and FMOC25_2 were treated with increasing doses of SHP099 together with either selumetinib or trametinib for one week followed by cell viability measurement. For synergy assessment, the highest single agent (HSA) synergy model was applied, using the R-package SynergyFinder. Results are presented as 2D contour plots where synergistic inhibition of cell viability is indicated by red color and antagonism by green color.

Supplementary Figure 8

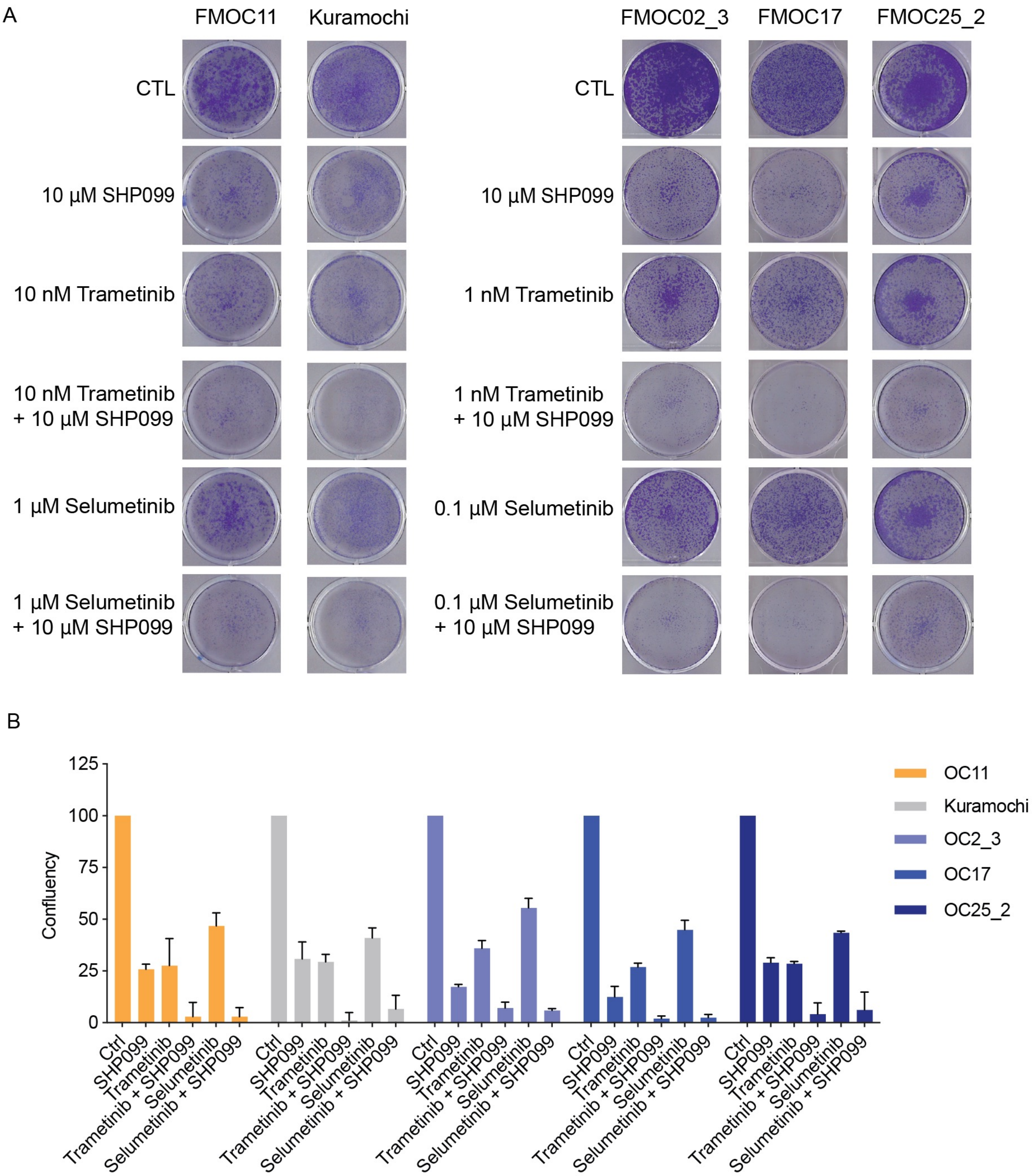

Supplementary Figure 8. SHP2 inhibitor SHP099 in combination with MEK inhibitors trametinib and selumetinib synergistically inhibits EOC PDCs cell growth in colony formation assay. A) Representative images of long-term colony formation assay with OC PDCs and control cell line Kuramochi. Cells were seeded at low density and cultured in the presence of DMSO, SHP099 (10 µM), trametinib (1 or 10 nM), selumetinib (0.1 or 1 µM), or SHP099+trametinib and SHP099+selumetinib combination for 12-14 days, and stained with crystal violet solution for imaging. Representative images from three independent experiments are shown. B) Colonies were quantified by ImageJ using the ColonyArea plugin. Shown are percentage inhibition by comparison with the control (DMSO treated cells). Shown results are representative of at least two independent experiments. Data are presented as means ± SEMs.

Supplementary Figure 9

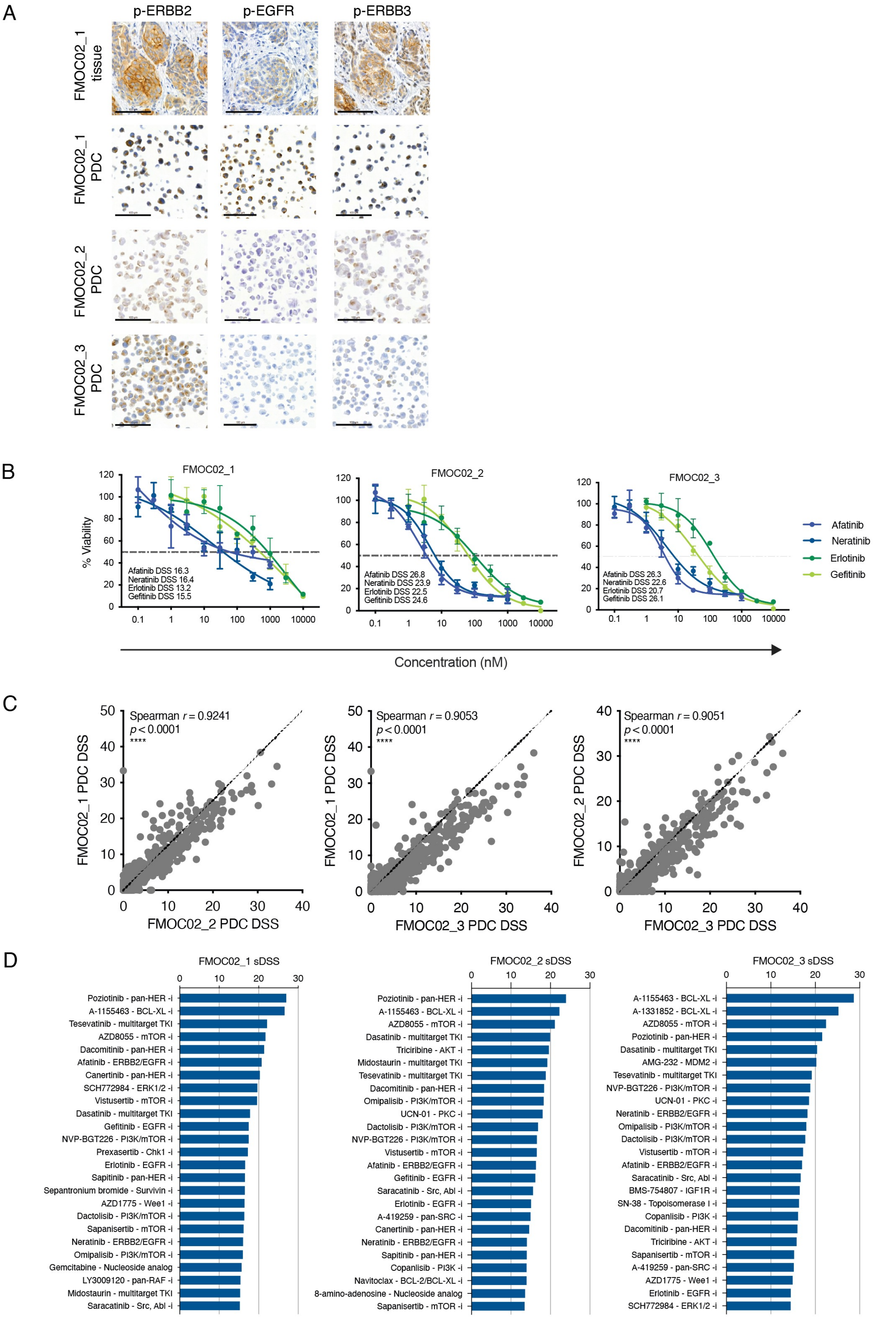

Supplementary Figure 9. A) Expression of p-ERBB2, p-EGFR and p-ERBB3 in FMOC02_1 tumor tissue (peritoneal metastasis from 2014) and three PDCs was assessed by immunohistochemistry (IHC). Micrograph pictures were taken at a 20x magnification. Scale bar, 100 µm. B) Dose response curves of three FMOC02 PDCs to ERBB2/EGFR inhibitors afatinib and neratinib and to EGFR inhibitors erlotinib and gefitinib tested in 3D condition as spheroids. C) Comparison of drug sensitivities between three FMOC02 PDCs (FMOC02_1, FMOC02_2 and FMOC02_3) to FO5 drug library. D) Top 25 most effective drugs for FMOC02 PDCs presented as selective DSS.
